# The 3-Hydroxy-2-Butanone Pathway Is Required for *Pectobacterium carotovorum* Pathogenesis

**DOI:** 10.1371/journal.pone.0022974

**Published:** 2011-08-18

**Authors:** Maria del Pilar Marquez-Villavicencio, Brooke Weber, R. Andrews Witherell, David K. Willis, Amy O. Charkowski

**Affiliations:** 1 Department of Plant Pathology, University of Wisconsin-Madison, Madison, Wisconsin, United States of America; 2 Vegetable Crops Research Unit, Agricultural Research Service, United States Department of Agriculture, Madison, Wisconsin, United States of America; University of Wisconsin-Milwaukee, United States of America

## Abstract

*Pectobacterium* species are necrotrophic bacterial pathogens that cause soft rot diseases in potatoes and several other crops worldwide. Gene expression data identified *Pectobacterium carotovorum* subsp. *carotovorum budB*, which encodes the α-acetolactate synthase enzyme in the 2,3-butanediol pathway, as more highly expressed in potato tubers than potato stems. This pathway is of interest because volatiles produced by the 2,3-butanediol pathway have been shown to act as plant growth promoting molecules, insect attractants, and, in other bacterial species, affect virulence and fitness. Disruption of the 2,3-butanediol pathway reduced virulence of *P. c.* subsp. *carotovorum* WPP14 on potato tubers and impaired alkalinization of growth medium and potato tubers under anaerobic conditions. Alkalinization of the milieu via this pathway may aid in plant cell maceration since *Pectobacterium* pectate lyases are most active at alkaline pH.

## Introduction

Plant associated *Enterobacteriaceae* commonly produce acetoin (3-hydroxy-2-butanone; 3H2B) from pyruvate, which is an end product of fermentation of glucose or other carbon sources through the Embden-Meyerhof (EM) pathway [1], [2], whereas related animal pathogens produce acid and sometimes gas through mixed acid fermentation. The Voges-Prokauer reaction, which measures production of 3H2B, has been used in microbiology lab classes and taxonomic schemes for decades to differentiate environmental *Enterobacteriaceae* from coliform bacteria, which are used as indicators of fecal contamination [3]. In the 3H2B pathway, α-acetolactate synthase condenses two molecules of pyruvate into α-acetolactate, which is then converted into 3H2B (reviewed in [4]). Nonenzymatic oxidative decarboxylation can also convert α-acetolactate into diacetyl. The enzyme 2,3-butanediol dehydrogenase transforms 3H2B into 2,3-butanediol (23B) and also transforms diacetyl into 23B. The genes encoding the 3H2B pathway enzymes are commonly referred to as *bud* genes. In addition to the plant-associated *Enterobacteriaceae*, such as *Pectobacterium*, *Dickeya*, *Erwinia*, *Enterobacter*, *Serratia*, and *Klebsiella*, many other genera of Gram-positive and Gram-negative bacteria, such as *Pseudomonas*, *Streptomyces*, *Staphylococcus*, *Bacillus* and *Clostridium* species, use this pathway to produce 3H2B and its reductive state analog 23B.

The 3H2B pathway alkalinizes growth media during fermentation, thereby promoting growth of bacteria unable to cope with acidic conditions. For example, acidic conditions are lethal to *Vibrio cholerae* and acquisition of the 3H2B pathway by the El Tor biotype appears to have made it a better intestinal and environmental colonist [5]. Since 3H2B and 23B can be metabolized by some bacterial species after glucose and other readily fermented carbon sources are used, it also serves as an energy storing strategy [6], [7], [8]. The 3H2B pathway may also reduce fitness in some environments; a *Serratia plymuthica budB* mutant is more virulent on carrot slices than wild type, even though it does not grow as well in rich medium as wild type [9].

3H2B and the related compound 23B function as signal molecules for both plants and animals. In humans, 23B alleviates lung inflammation via interaction with the NF-κB pathway and is thought to play a role in pathogenesis of lung-infecting bacteria [10]. These molecules have other unfortunate connections to lung disease. 3H2B and diacetyl are commonly used in food to give a buttery flavor and diacetyl is thought to be responsible for “popcorn worker's lung” [11]. 3H2B and 23B also act as pheromones or kairomones and attract a wide variety of insects, including sap beetles (*Carpophilus hueralis*) [12], lygus bugs (*Lygus* sp.) [13], cockroaches (*Nauphoeta cinerea*) [14], [15], Melanesian rhinoceros beetles (*Scapanes australis*) [16], sorghum chafers (*Pachnoda interrupta*) [17] and Mexican fruitflies (*Anastrepha ludens*) [18]. The attraction mechanism has only been described for fruit flies. These insects normally avoid CO_2_, which is emitted by ripening fruit, but 23B inhibits antenna neurons sensitive to CO_2_ [19]. These compounds can affect insect reproduction as well. For example, 3H2B increases the time it takes for female cockroaches to reach parturition, which increases their lifespan, but decreases the number of male offspring initially produced [20], [21].

In *Arabidopsis* plants, 3H2B and 23B promote plant growth, drought tolerance, and defense against pathogens. Experiments with plant growth promoting *Bacillus* species showed that volatiles produced by *Bacillus* were responsible for increased growth of *Arabidopsis* plants and these volatiles were later identified as 3H2B and 23B [22]. The plant growth promoting *Bacillus* was unable to affect Arabidopsis *ein2* and *cre1* mutants, which are impaired in ethylene and cytokinen sensing, respectively, suggesting that 3H2B and 23B act on plants through these pathways. Bacterial 3H2B and 23B also appear to act as microbe-associated molecular patterns (MAMP). These volatiles induce systemic resistance in Arabidopsis [23] and also cause Arabidopsis stomata to close [24].

The 3H2B pathway is present in the *Enterobacteriaceae* plant pathogens *Pectobacterium* and *Dickeya* [25]. These genera are found worldwide in water, soil, and plants and cause wilt, necrosis and maceration symptoms in potato, as well as numerous other vegetable and ornamental plants [26]. Both genera produce acetoin in culture and volatile products of the HB pathway have been detected when assaying carrots and potatoes inoculated with *Pectobacterium carotovorum* subsp. *carotovorum* (*P. c.* subsp. *carotovorum*) [27], [28]. Volatile products may also play a role in vectoring soft rot pathogens since insects appear to play important roles in both *Pectobacterium* and *Dickeya* life cycles [29], [30], [31], [32], [33], [34], [35], [36], [37], [38], [39], [40], [41].

These soft rot pathogens secrete multiple plant cell wall degrading enzymes during pathogenesis, including pectate and pectin lyases, polygalacturonases, cellulases and proteases [42], [43]. The pectate lyses are crucial for disease development. Bacterial pectate lyases tend to have a pH optimum of over 8 (for example, see [44], [45]), while the plant host apoplast pH is typically 5 to 6.5 [46]. Thus, raising the local pH is a necessary step in soft rot pathogenesis [47] and it is possible that the 3H2B pathway plays a role in this step of pathogenesis. We recently found that the genes encoding this pathway, including *budA*, *budB*, and *budC*, are highly expressed during *P. c.* subsp. *carotovorum*-potato stem and tuber interactions (Marquez-Villavicencio et al., in preparation) and RT-qPCR confirmed that *budB* is differentially expressed in potato stems compared to potato tubers. Here we describe the effects of a *budB* mutation on *P. c.* subsp. *carotovorum* virulence in potato tubers.

## Materials and Methods

### Bacterial strains and growth conditions

Bacterial strains used in this study are listed in Table 1. *P. c.* subsp. *carotovorum* strains were grown in Luria-Bertani medium (LB) at 37°C. When required, antibiotics were used at the following concentration: ampicillin (100 µg/ml), spectinomycin (50 µg/ml), and kanamycin (50 µg/ml).

**Table 1:** Bacterial strains and plasmids used in this study.

Strains/plasmids	Relevant characteristics	References
***E.coli***		
DH5α	*supE44 ΔlacU169 (Δ80lacZΔM15) hsdR17 recA1EnA1 gyrA96 thi-1 relA1*	Clontech
***P. c.*** ** subsp. ** ***carotovorum***		
WPP14	Wild type strain isolated from an infected potato stem in Wisconsin, 2001	[75], [76]
WPP502	Km^R^; Δ*budB::km* derivative of WPP14	This work
WPP504	Km^R^, Ap^R^, derivative of WPP502 harboring pTA::*budAB*	This work
**Plasmids**		
pCPP50	Ap^R^pINIII_113_-A2-based expression vector	[77]
pGEM®-T Easy	Ap^R^, *lacZ′*, cloning vector	Promega
pHP45ΩSp	Ap^R^, Sp^R^/Sm^R^, template plasmid carrying Sp^R^ cassette	[78]
pKD4	Km^R^, template plasmid carrying *kan* cassette	[79]
pTAΔ*budB*_AD	Ap^R^, 1.6-kb upstream of *budB* fused by a *Hind*III site to 1.6-kb downstream of *budB* site in pGEM®-T Easy	This work
pTAΔ*budB*_AD::*km*	Ap^R^, Km^R^, 4.8-kb fragment containing *budB*_AD cut with *Hind*III and fused to *kan* cassette in pGEM®-T Easy	This work
pTA*budAB*	Ap^R^, 2.6-kb fragment containing the *budAB* operon and its promoter region in pGEM®-T Easy	This work

### Plant growth conditions and inoculation methods for plants used for bacterial mRNA isolation

Certified seed potatoes (*Solanum tuberosum* subsp. *tuberosum* var. Superior) were used for experiments where bacterial RNA was isolated from plant samples. Potato plants were grown in a growth chamber at 26°C with a 12 hour photoperiod and were not fertilized since the tuber provides enough nutrition for young potato plants. Stems of 4-week old plants were inoculated by stabbing a needle and injecting 10 µl of a bacterial suspension of WPP14 (0.3 OD_600_, equivalent to 1.5×10^8^ CFU/ml). To prepare bacterial suspensions, cells from 24 hour-old bacterial cultures grown on LB agar medium incubated at 37°C were suspended in water. Each potato stem was inoculated in five different sites approximately 3 cm apart. After inoculation, plants were placed into plastic bags and incubated at 26°C for 13.5 hours. Based on growth curve analysis, the bacteria reached log phase growth between 10 and 15 hours after inoculation into stems (data not shown).

For experiments where bacterial RNA was isolated from inoculated tubers, the potato tubers (*S. tuberosum* subsp. *tuberosum* var. Superior) were surface sanitized by soaking them in 0.5% bleach for 10 minutes. The tubers were rinsed with distilled water and allowed to dry at room temperature for 24 hours. The tubers were inoculated in at least 15 sites per tuber with 10 µl of a bacterial suspension of WPP14 (1.5×10^8^ CFU/ml). Tubers were then placed into plastic bags and incubated at 26°C for 10 hours. Based on growth curve analysis, the bacteria reach log phase growth between 10 and 15 hours after inoculation into stems (data not shown).

### Bacterial RNA isolation

Approximately 90 stem and 75 tuber inoculations sites, respectively, were sampled for bacterial RNA isolations. Thirty stem sections of approximately 1 cm each or 25 1-cm cubes of tuber were placed into a cooled sterile RNAse-free mortar that was pre-filled with 17.5 ml of DEPC water and 2.5 ml of ice cold EtOH/phenol stop solution (5% water-saturated phenol pH<7.0) to stabilize cellular RNA and stop degradation [48], [49]. Plant samples were gently ground with a sterile RNAse-free pestle. Supernatants were collected in two 15 ml falcon tubes and RNA was isolated with a hot SDS/hot phenol protocol [48], [49]. Briefly, bacterial supernatants were harvested by centrifugation 8,200 g for 2 minutes at 4°C. The supernatant was aspirated and the pellet was frozen in a dry ice-alcohol bath. The pellets were resuspended in 1.6 ml of lysis buffer (TE: 10 mM Tris - 1 mM EDTA, pH 8.0 and 0.5 mg/ml Ready-Lyse™ Lysozyme Solution (Epicentre Biotechnologies, Madison, WI) and 160 µl of 10% SDS solution, and then mixed and incubated at 65°C for 2 minutes. Then, 176 µl of 1 M NaOAc (pH 5.2) was mixed with the lysate. Equal volumes of lysate were transferred to RNase-free 2 ml microfuge tubes and mixed by inversion with 1 ml of water saturated phenol. The tubes were incubated in 65°C water bath for 6 minutes. Then the tubes were placed on ice for 2 minutes and centrifuged at 21,000 g for 10 min at 4°C. The aqueous layer was then transferred to a new 2 ml microfuge tube containing an equal volume of chloroform. Tubes were mixed by inversion, and then spun down at 21,000 g for 10 minutes at 4°C. The aqueous layer was split into equal volumes in 1.5 ml Eppendorf tubes and ethanol precipitated by adding 1/10 volume of 3 M NaOAc (pH 5.2), 1 mM EDTA (final concentration) and 2 volumes of cold 100% EtOH to each sample. Samples were mixed and placed at −80°C overnight. Bacterial RNA was pelleted by centrifugation at 21,000 g for 30 minutes at 4°C. The pellets were washed with ice cold 80% EtOH and spun down at 21,000 g for 10 minutes. The supernatant was removed and the pellets were air dried for 30 minutes in the fume hood. The pellets were resuspended in 10 µl of DEPC-treated water. All resuspended samples were pooled together (100 µl) and DNA contamination was removed by treatment with Turbo DNA-*free* (Ambion, Inc.) with the rigorous protocol and with 5 µl of enzyme. Additional sample purification was performed with RNeasy Mini Kits (Qiagen, Valencia, CA). Purity and concentration of the RNA was verified with a spectrophotometric analysis (NanoDrop ND-1000; NanoDrop technologies, Wilmington, DE). RNA integrity was measured with an Agilent 2100 Bioanalyzer (Agilent Technologies, Inc.) and the RNA was stored at −80°C until use.

### RT-qPCR

Quantitative real-time reverse transcriptase PCR (RT-qPCR) was used to validate the transcriptome profiling experiment on selected candidate genes. We followed the recently published MIQE guidelines for publication quality RT-qPCR [50]. Bacterial RNA was isolated as described above. Primers were designed based on the draft genome sequence of WPP14 with Beacon Designer software (Premier Biosoft International, Palo Alto, CA, U.S.A) and primer sequences are shown in Table 2. Primer efficiency was determined with a dilution series of *P. c.* subsp. *carotovorum* WPP14 chromosomal DNA. RNA samples were tested for residual DNA contamination with RT-qPCR and primers specific for the *proC* transcript in *P. c.* subsp. *carotovorum* WPP14. RNA samples that showed quantification cycle (C_q_) values greater than 30 cycles were considered to be sufficiently free of chromosomal DNA contamination for analysis. The synthesis of cDNA was performed with an iScript cDNA synthesis kit according to the manufacturer's instructions (Bio-Rad Laboratories, Hercules, CA, U.S.A.). Briefly, cDNA synthesis was performed with 250 ng of total RNA in 15 µl of DEPC-water plus 4 µl of 5× iScript reaction mix, which contains a blend of oligo dT and random hexamer primers and 1 µl of iScript reverse transcriptase. The cDNA synthesis mixture was incubated at 25°C for 5 min. followed by 42°C for 30 min., then 85°C for 5 min. Three independent biological replicate samples per experimental condition were completed for the cDNA synthesis. Two iScript reactions were performed for each RNA sample. For each cDNA sample, two 25 µl reactions were used as technical replicates. The amount of mRNA specific cDNA was determined with qPCR with the MyiQ detection system (Bio-Rad Laboratories). The PCR conditions were 95°C for 3 min; 40 cycles of 95°C for 10 s and 50°C for 45 s; and 1 cycle of 95°C for 1 min and 55°C for 1 min; followed by a dissociation curve with 80 cycles of 55°C for 10 s with a 0.5°C increase per cycle. Primer dimers and the presence of a single product per reaction were evaluated with the dissociation (melt) curve analysis in the MyiQ detection system software.

**Table 2:** RT-qPCR primer sequences and efficiency.

Gene name	Primer sequence (5′→3′, forward to reverse)	Efficiency (%)^A^
*budB*	TTGAATCTGCTGATGAAC	
	CAATGGTTATCGGAATAATC	102.8
*dspE*	GTCCTATACCAACCTCAG	
	GCAACGAAGAGAACAAAT	98.8
*ffh*	TGGAAACATTGGCAGAGC	
	GACTAACAAGACATCGTAGAAC	105
*hrcC*	GCCTATTCTGCCGAACAA	
	GCACCAAATCCACACCAT	99.9
*pelB*	CTCCGTAACAACAACATT	
	GTACTCTTCCAGTCATCT	102.3
*proC*	GTGCGAATTATGCCAAAC	
	TTATCTGCCTGACTGACG	100.7

A : Primer efficiencies were calculated from dilution curve with the MyiQ Cycler software.

For absolute quantification, the target transcript amount in each sample was determined with a standard curve of *P. c.* subsp. *carotovorum* WPP14 DNA. The mean of the starting quantity (SQ) ratio for each target mRNA was internally normalized with the absolute expression mean values of four reference transcripts: *ffh*, *dspE*, *pelB*, and *hrcC*. The SQ value of each reference RNA was determined with a standard curve of *P. c.* subsp. *carotovorum* WPP14 DNA. These four RNA were validated as stable reference transcripts under our experimental conditions with the Excel-based software program BestKeeper [49], [50]. We determined the mean relative expression ratio (RER) of the *budB* transcript in tuber tissue relative to stem by first normalizing the target transcript in each sample to each of the four reference transcripts and then determining the ratio of expression in each of the three stem and tuber samples relative to the mean of expression in stem tissue. The means of the stem and tuber ratios were analyzed via a two-tailed unpaired *t*-test with a 95% confidence interval with Prism 5.0a software (GraphPad Software, Inc. La Jolla, CA, U.S.A).

### Mutants and plasmid constructions

To construct plasmids for allelic exchange mutagenesis of *budB*, 1.6-kb flanking regions of *budB* were PCR-amplified with primers sets (University of Wisconsin Biotechnology Center DNA Synthesis Facility, Madison, WI) listed in Table 3. Primers were used to amplify a 3.2-kb fusion of flanking regions amplicons. A unique *Hind*III enzyme site was added into the primers, allowing cleavage and ligation of the antibiotic resistance gene *kan* between the flanking regions. The fusion PCR product was cloned into pGEM®-T Easy vector (Promega, Madison, WI), producing pTAΔ*budB*_AD::km vector. This plasmid was electrotransformed into wild-type WPP14 for allelic exchange mutagenesis following the methods described by Ried and Collmer [51]. The mutation was confirmed by PCR-amplifying the mutated region and sequencing the PCR fragment. To complement the Δ*budB* mutation, the promoter region and coding regions of *budA* and *budB* were PCR amplified and cloned into the pGEM®-T Easy vector and the resulting plasmid was electrotransformed into Δ*budB*. Transformation, restriction endonuclease digestion and other DNA manipulations were performed essentially as described in Sambrook and Russell [52].

**Table 3:** Oligonucleotides used in this study for cloning and mutagenesis.

Sequence (5′→3′)	Restriction sites	Amplified region
TTAATACAGGCGAGAGATTCC	**—**	1.6-kb upstream
CGTACTCTGCGAAGCTTCCGACATGACTCCGATGAAATGACTAGCTC	*Hind*III	*budB*
		
GGAAGCTTCGCAGAGTACGTATATCAGGTGCTTATCCGCC	*Hind*III	1.6-kb downstream
AAACAT TAATCACGGACCAATATC	*—*	*budB*
		
ATGGAAAAGTCCACCGAACAGCA	*—*	0.716-kb
CAGATCAGCACGCTAATATTCAGATTC	*—*	*budB* gene
		
AGATAACGTAGCTCCATTTACGTGA	*—*	2.6-kb *budA::budB*
TCAGATCAGCACGCTAATATTCAGATTC	*—*	with native promoter

### Voges-Proskauer test

To verify a disruption in the acetoin pathway, we used the Voges-Proskauer test, which determines whether 3H2B is present [3]. A single colony of WPP14 wild- type, Δ*budB*, and the complemented strain of Δ*budB* grown in LB agar medium, were transferred to 5 ml of methyl red-Voges Proskauer buffered glucose broth (BD Difco™, NJ) amended with appropriate antibiotics and the culture were incubated at 37°C. The presence of 3H2B, was monitored at 24 and 48 hours of incubation. For this, 1 ml of each bacterial culture was transferred to a sterile glass tube and 15 drops of BBL Voges-Proskauer reagent A (5% wt/vol alpha-naphthol in absolute alcohol) (BD, Franklin Lakes, NJ) and 5 drops of BBL Voges-Proskauer reagent B (40% wt/vol potassium hydroxide in distilled water) were added. The mixture was stirred vigorously and the development of a red color, which indicates a positive test, was monitored for 30 minutes at room temperature.

### Measurements of pH under *in vitro* and *in vivo* conditions

To evaluate differences in pH between WPP14 wild-type and Δ*budB* after growing in a glucose-rich medium, single colonies grown in LB agar medium, were transferred to 3 ml of SOC (glucose-containing rich medium) and incubated at 30°C for 20 hours. The pH was then measured with EMD colorpHast pH strips (4 to 7 and 6.5 to 10 pH range) (Merck KGaA, Darmstadt, Germany). Measurements were done in duplicate and the experiment was repeated twice. To evaluate differences in pH between WPP14 wild-type and WPP14Δ*budB* during disease in potato tubers, slices of surfaced-sanitized tubers (cv. Yukon Gold) of 1.5 cm thickness were inoculated with 10^6^ CFU bacteria in two sites on each slice. For incubation under aerobic conditions, individual potato slices were placed into sterile plastic petri dishes, sealed with parafilm and incubated at room temperature. For incubation under anaerobic conditions, potatoes slices in petri dishes or whole potato tubers, were placed into a GasPak 100 polycarbonate jar (BD, Franklin Lakes, NJ). GasPak™ Plus envelopes (BD, Franklin Lakes, NJ), which are disposable hydrogen and carbon dioxide generators, were used to create an anaerobic atmosphere. The pH in potato slices after inoculation was monitored with the following pH indicator solutions: bromocresol purple (0.1%), which is yellow when the pH is below 5.2 and purple when the pH is above 6.8, and phenol red (0.1%), which is yellow when the pH is below 6.4 and red violet when the pH is above 8.2 [53]. Measurements were done in duplicate and the experiment was repeated twice.

### Virulence assays and bacterial growth curves in potatoes

Differences in macerated tissue produced by wild type and mutants strains were evaluated with a potato tuber test. For this, potato tubers (cv. Yukon Gold) were surface-sanitized for 10 min with 10% bleach, rinsed thoroughly and allowed to air dry. For inoculation, bacterial strains were grown overnight on LB agar medium and suspended in sterile water. For each biological replicate of the experiment, 10 tubers were stabbed approximately 1.5 cm deep with a pipette tip and 10 µl of a 10^8^ CFU/ml bacterial suspension was placed into the wound. Negative controls were inoculated with sterile water. The potato tubers were placed into plastic bags and the bags were sealed to maintain moisture and create a low oxygen environment. They were then randomized in plastic trays and incubated at 28°C for 3 days. Diseased tubers were cut open and macerated tissue was scooped out and weighed. The experiment was repeated twice. Statistical analysis was performed with a two-tailed *t*-test with 95% confidence interval with Prism version 5.0a (GraphPad Software, Inc., La Jolla, CA, U.S.A).

To determine if mutations affected bacterial growth in potato stems and tubers, bacterial populations were monitored. Surface sanitized potato tubers (cv. Superior) were stab-inoculated by placing 10 µl of a 10^7^ CFU/ml suspension of WPP14 wild-type or Δ*budB* into 1-cm-deep wounds made with a pipette tip. Negative controls were inoculated with sterile water. Tubers were placed individually into plastic bags, the bags were sealed, and the tubers were incubated at 28°C. Bacterial growth was monitored at 0, 24, 48 and 72 hours after inoculation. Approximately 1 g of potato tissue was recovered from the inoculation site with a number 5 cork borer, weighed, and homogenized in sterile water in sterile polystyrene tubes (12×75 mm) with a sterile plastic pestle. Bacterial populations in the homogenates were determined by dilution plating on LB agar medium, incubating the plates at 37°C for 24 hours, and counting the resulting colonies. Five replicates were used to calculate means and standard errors. Statistical analysis in each time point was performed with a two-tailed *t*-test with 95% confidence interval using Prism version 5.0a (GraphPad Software, Inc., La Jolla, CA, U.S.A).

### Assaying acetoin and 2, 3-butanediol for effects on potato plantlet growth

Tissue culture plantlets (cv. Atlantic) obtained from the Wisconsin Seed Potato Certification Laboratory were used to examine twenty-six treatments consisting of 3H2B and 23B separately and in combination (Table 4). 3H2B (Acetoin A17951) and 23B (2, 3-butanediol B84904) were obtained from Sigma-Aldrich. Different concentrations of each compound were made with the solvent dichloromethane (D37-500, Fisher) and sterilized with a 0.2 µM nylon filter into sterile centrifuge tubes. Lanolin (S80047-1, Fisher), which has been reported to reduce the initial burst of 3H2B and 23B [23] was added at a rate of 80 mg/ml to a subset of the treatments to determine if the addition of lanolin affected shoot growth.

**Table 4:** Twenty-six treatments used to examine 3H2B (acetoin) and 23B (2, 3-butanediol) separately and in combination at various concentrations, in the presence or absence of lanolin, and at two application rates.

Treatment	Compound	Lanolin addition	Compound conc.	Rate applied
1	2,3-butanediol	Yes	10 µg/ml	10 µl
2	2,3-butanediol	Yes	100 ng/ml	10 µl
3	2,3-butanediol	Yes	1 ng/ml	10 µl
4	2,3-butanediol	Yes	10 pg/ml	10 µl
5	2,3-butanediol	No	10 µg/ml	10 µl
6	2,3-butanediol	No	100 ng/ml	10 µl
7	2,3-butanediol	No	1 ng/ml	10 µl
8	2,3-butanediol	No	10 pg/ml	10 µl
9	Acetoin	Yes	1 mg/ml	10 µl
10	Acetoin	Yes	10 µg/ml	10 µl
11	Acetoin	Yes	100 ng/ml	10 µl
12	Acetoin	Yes	1 ng/ml	10 µl
13	Acetoin	No	1 mg/ml	10 µl
14	Acetoin	No	10 µg/ml	10 µl
15	Acetoin	No	100 ng/ml	10 µl
16	Acetoin	No	1 ng/ml	10 µl
17	Combination^A^	No	10 µg/ml, 1 mg/ml	10 µl
18	Combination	No	100 ng/ml, 10 µg/ml	10 µl
19	Combination	No	1 ng/ml, 100 ng/ml	10 µl
20	Combination	No	10 pg/ml, 1 ng/ml	10 µl
21	Combination	No	10 µg/ml, 1 mg/ml	20 µl
22	Combination	No	100 ng/ml, 10 µg/ml	20 µl
23	Combination	No	1 ng/ml, 100 ng/ml	20 µl
24	Combination	No	10 pg/ml, 1 ng/ml	20 µl
25	H_2_0	No	NA	10 µl
26	Solvent control	No	NA	10 µl

A In combination treatments, the applied rate was added per compound with the 23B concentration listed first.

Nine single nodal cuttings were spaced evenly in a culture vessel (Magenta GA7 cubes, Magenta Corp.) containing 75 ml of MS propagation medium (pH 5.85) for each treatment [54]. Under sterile conditions, a 1 cm filter paper was randomly placed in each cube and 10 µl or 20 µl of each compound (individually or in combination) were dispensed as specified per treatment. Two days after initiating the plantlets, shoot height was measured (in cm) every two days for 10 days. For the duration of the experiment, the plantlets were grown in a walk-in growth room set to a 16-h photoperiod under cool white fluorescent lamps that provided an average photosynthetically active radiation (PAR) of 200 µmol m^−2^ s^−1^. The temperature was maintained at 19°C with a relative humidity of 25%. The experiment was replicated three times with the culture vessels sealed with micropore tape, which allows volatiles, such as ethylene to disperse, and three times with tightly sealed culture vessels.

An analysis of variance (ANOVA) was performed with PROC MIXED in SAS® 9.2 (SAS Institute, Cary, NC). Models were fit both for reps combined and separately. PROC GLM was also performed to compare shoot height means per treatment with the Tukey multiple comparison correction. Means were given a letter code to determine significance at a *P* = 0.05 level.

## Results

### *P. c.* subsp. *carotovorum budB* is expressed at higher levels during bacterial growth in tubers than during growth in stems

**Figure 1:**
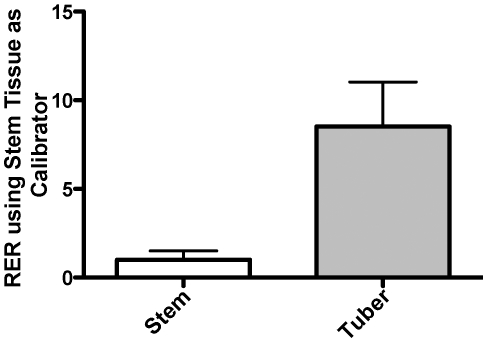
*P. c.* sp. *carotovorum budB* transcript is differentially expressed in potato tubers and stems. Relative expression of the *budB* transcript in potato stems and tubers was determined with four reference mRNA (*ffh*, *dspE*, *pelB*, and *hrcC* - Table 2). The mean relative expression ratio (RER) of the *budB* transcript in tubers was 8.5-fold elevated compared to stem tissue, which was used as the calibrator. The difference was found to be significant at a 95% confidence interval by *t*-test using Prism software (Mac version 5.0d, GraphPad Software, Inc.) with a *P*-value = 0.0425. Error bars represent standard error of the mean.

The *budAB* gene cluster first caught our attention when we noted that it was highly expressed in tubers in our microarray experiments. Details on the data obtained in our microarray experiment will be reported elsewhere (Marquez-Villavicencio et al., in preparation). We used absolute quantification of mRNA relative to four reference transcripts to confirm that one of the genes in this cluster, *budB*, was highly expressed during bacterial infection of tubers relative to stems. We determined the relative expression ratio (RER) with the mean values of the absolute amounts of four reference transcripts: *ffh*, *dspE*, *pelB*, and *hrcC* as previously described [48], [55]. The target RNA was normalized to each reference RNA by dividing the absolute amount of target RNA by the amount of reference RNA. To determine the relative expression ratio of the target gene in tubers relative to stems, the normalized target RNA ratio was divided by the average of the normalized ratios of three stem samples. The average of the three stem normalized ratios is designated the “calibrator” since the variation of all samples, including the individual stem samples, is determined relative to this value. Using four reference transcripts resulted in four mean RER values for each RNA sample relative to the calibrator. The four mean RER values for each of the stem and tuber samples were treated as replicates and statistically analyzed by *t*-test. The Prism software (GraphPad Software, Inc.) averages the four RER values for each of the three stem or tuber RNA samples before performing the *t*-test analysis. Our RT-qPCR results showed that *budB* expression was 8.5- fold higher in tubers than in stems (Fig. 1).

### The *P. c. subsp. carotovorum* WPP14 *budB* mutant does not produce detectable 3H2B in culture medium

**Figure 2:**
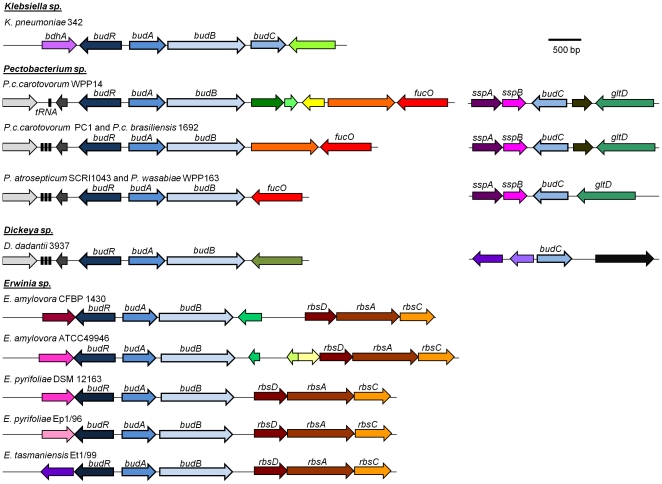
Operon structure of *bud* genes in *Klebsiella*, *Pectobacterium*, *Dickeya*, and *Erwinia* species. Sequences were retrieved from ASAP [66]. Genes are indicated by arrows, with the direction of the arrowhead indicating the direction of the gene. Genes shaded with the same color are homologous and unlabeled genes have no known function. *budA* is an alpha-acetolactate decarboxylase, *budB* is an acetolactate synthase, *budC* is an acetoin reductase and *budR* is a LysR-family transcriptional regulator. In *Klebsiella* and *Enterobacter* (not shown), *budABC* are in one operon. The locus of the *budC* gene is found elsewhere in the chromosome in *Pectobacterium* and *Dickeya*. The *Erwinia* species lack *budC*.

*P. c. subsp. carotovorum* WPP14 encodes genes homologous to *budAB*, *budC* and *budR*, which in other enteric bacteria are involved in the production of acetoin and 2,3-butanediol [4], [7]. The catabolic α-acetolactate synthase (*budB*) and α-acetolactate decarboxylase (*budA*), are found in the same locus and a third gene, butanediol dehydrogenase (*budC*) is located elsewhere in the chromosome, as described in *Bacillus subtilis* [56] (Fig. 2). This differs from other *Enterobacteriaceae* such *Klebsiella* and *Enterobacter* in which the three enzymes are found in one operon [57], [58]. In the *Enterobacteriaceae*, the transcriptional regulator *budR* is located in the same locus as the *budAB* operon, but is divergently transcribed. Other soft rot pathogens, including *P. atrosepticum* SCRI1043, *P. carotovorum* PC1, *P. carotovorum* subsp. *brasiliensis* 1692, *P. wasabiae* WPP163, *Dickeya dadantii* 3937 have the same operon structure as *P. c. carotovorum* WPP14. Members of the *Erwinia* genus, such *Erwinia amylovora* CFBP1430 and ATCC 49946, *Erwinia pyrifoliae* DSM 12163 and Ep1/96 and *Erwinia tasmaniensis* ET1/99, have the same *budRAB* operon structure as *P. c. subsp. carotovorum* WPP14, but all of these *Erwinia* species lack *budC*, suggesting that they produce 3H2B, but not 23B. In the plant associated *Enterobacteriaceae*, the *budRAB* locus does not appear to be located in a conserved region of the chromosome since the genes flanking this locus vary among strains and species.

To test whether the *budAB* operon is required for 3H2B production, a *budB* deletion mutant was constructed. To verify a disruption in the butanediol pathway, a qualitative Voges-Proskauer test was used to detect the presence of acetoin through the formation of a red-colored compound in the medium [3]. While wild type WPP14 and the complemented Δ*budB* mutant strains turned red after adding alpha-naphthol and potassium hydroxide to the medium in which they were grown, the Δ*budB* culture did not. This result was observed at both 24 and 48 hours after incubation.

### *P. c.* subsp. *carotovorum* WPP14 *ΔbudB* is unable to alkalize culture medium or inoculated potato tubers

Activity of the 3H2B pathway is known to result in medium alkalization in other species [5]. Wild type WPP14 cultures grown in SOC medium reached pH 7.7 after 20 hours of incubation, whereas the WPP14Δ*budB* culture remained at pH 5.0. The complemented Δ*budB* mutant strain was partially restored, with a culture pH of 7.1. Uninoculated SOC medium remained at pH 7.0.

**Figure 3:**
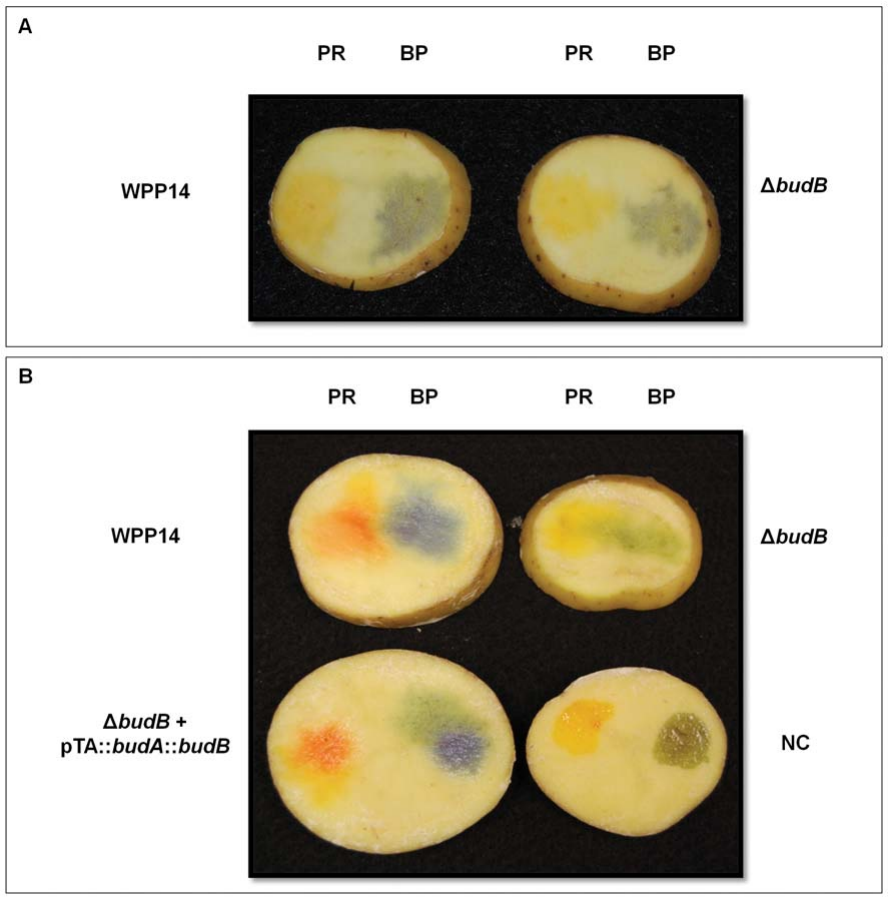
The *P. c.* subsp. *carotovorum budAB* operon affects bacterial modification of pH in tubers slices that are incubated anaerobically. Potato slices were inoculated with 10^6^ CFU and incubated under aerobic (**A**) and anaerobic conditions (**B**) for 20 hours at room temperature (∼22°C). **PR**: pH indicator phenol red (yellow at pH below 6.4 and red violet at pH above 8.2), **BP**: pH indicator bromocresol purple (yellow at pH below 5.2 and purple at a pH above 6.8).

The pH of diseased tuber slices was measured after incubation in aerobic and anaerobic conditions to determine if *budB* also affected the pH of the milieu during plant-microbe interactions. After 6 or 20 hours of aerobic incubation, no differences in pH around the site of inoculation were observed between wild type and the Δ*budB* mutant; both remained below 6.4 (Fig. 3). However, when potato slices were incubated in an anaerobic chamber, a difference in pH between wild type and Δ*budB* were seen at both 6 and 20 hours after incubation. While the tissue inoculated with wild type WPP14 reached a pH above 6.8, the *budB* mutant remained below pH 6.4. Expression of *budAB* from a plasmid in the mutant strain restored the wild type phenotype (Fig. 3).

### The *P. c.* subsp. *carotovorum* WPP14 *budB* mutant was reduced in virulence in potato tubers

**Figure 4:**
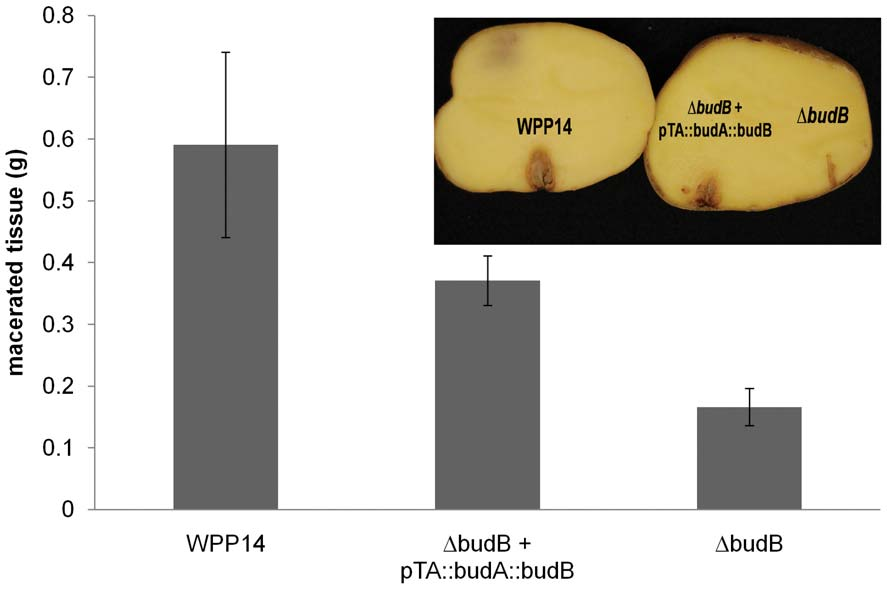
WPP14 Δ*budB* is reduced in virulence in potato tubers. Potato tubers (n = 10) were inoculated with 10^6^ CFU, placed into plastic bags and incubated at 28°C for 3 days. After incubation, macerated tissue was scooped out and weighed. The graph shown is from one experiment. This experiment was repeated twice with similar results. Error bars indicate standard error.

**Figure 5:**
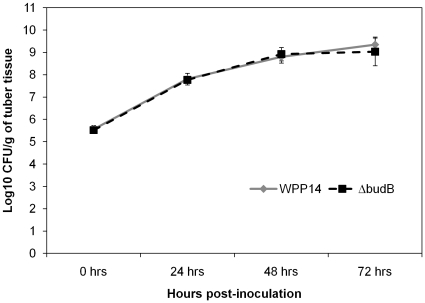
WPP14Δ*budB* is not impaired in growth in potato tubers. Bacterial suspensions containing 10^5^ CFU were stab-inoculated into potato tubers (cv. “Superior”) (*n* = 5). Tubers were placed into plastic bags and incubated at 28°C. Bacterial population was determined every 24 hrs after inoculation. Error bars indicate standard error. The graph shows data from one of two experiments, neither of which showed significant difference in bacterial growth.

Because a functional *budB* gene was required for alkalinization of both SOC medium and potato tissue, we hypothesized that this gene was also important for virulence in potato tubers. In two independent experiments, the *budB* mutant produced significantly less macerated tissue than wild type (Fig. 4, two-tailed *t*-test, *P*<0.001). Expression of *budAB* from a plasmid in the mutant strain partially restored the wild type phenotype (Fig. 4). To determine if the reduction in virulence was due to reduced ability of the bacteria to multiply in lesions, the bacterial population in the symptomatic part of the potato tuber was monitored. The mutant and the wild type strains were inoculated in potato tubers both grew to ∼10^9^ CFU/g of tissue after 3 days of incubation (Fig. 5). No statistically significant difference in bacterial growth was detected at any time point of bacterial growth curve by a two-tailed *t*-test. Thus, the mutant is not defective in growth in the sense that it was able to grow in potato tuber at the inoculation site to the same density as wild type. However, the reduced ability of the mutant to macerate potato tissue and spread beyond the inoculation site results in an overall reduction in bacterial growth in tubers.

### Acetoin (3H2B) and 2,3-butanediol (23B) do not affect potato growth in tissue culture

3H2B suppresses the ethylene response in Arabidopsis [22], [23] and insensitivity to ethylene in both tobacco and Arabidopsis increases susceptibility *P. c.* subsp. *carotovorum* [59]. Ethylene accumulation in tissue culture vessels is a well-known cause of poor vigor and the reason that culture vessels are sealed with micropore tape to allow for ventilation, while still maintaining a sterile environment for tissue culture plantlets. To determine if 3H2B or 23B affected potato growth, we treated nodal cuttings of potato with these compounds in tightly sealed vessels or in vessels sealed with micropore tape. We were unable to detect any significant effect of 3H2B or 23B on growth of potato plantlets (data not shown).

## Discussion

Relatively little is known about global gene expression of bacterial plant pathogens during pathogenesis. We became interested in the *budAB* operon because it was initially among the most highly expressed *P. c.* subsp. *carotovorum* genes identified in bacterial tuber interactions through our microarray assay (Marquez-Villavicencio et al., in preparation). We used real-time RT-qPCR to confirm that *budB* is expressed during pathogenesis and also found it to be differentially expressed during infections of stems and tubers. In the plant pathogens *Pectobacterium atrosepticum* and *S. plymuthica*, the *budAB* operon is controlled by acyl-homoserine lactone quorum sensing [60], [61], and it is also controlled by pH in *S. plymuthica* [61]. In *V. cholerae*, this operon is controlled by multiple regulators and is induced by quorum sensing, acetate [62] as well as acidic pH and low oxygen levels [63].

We hypothesize that less oxygen is available in potato tubers than stems and that this alternate fermentation pathway could be responding to low oxygen levels, but since this operon is likely to be controlled by multiple stimuli, there are many other possibilities that could account for this difference. The *budAB* operon does play a role in *P. c.* subsp. *carotovorum* pathogenesis in tubers, perhaps in part due to the role of this gene cluster in alkalizing the local environment.

The Voges-Proskauer assay, which detects a product of the EM pathway, 3H2B (acetoin), has been used for decades to differentiate coliform bacteria, such as *Escherichia coli* and *Salmonella enterica* from other enterobacteria [3]. Although many plant pathogenic enterobacteria, including soft rot pathogens (*Pectobacterium* and *Dickeya*), necrotic vegetable pathogens (*Enterobacter*), and tree pathogens (*Erwinia amylovora*) encode this pathway, its role in plant pathogenesis has been little explored. We found that *budB*, which encodes α-acetolactate synthase, an essential enzyme in this pathway, is required by *P. c.* subsp. *carotovorum* for full virulence. Although others have reported that volatiles produced via this pathway promote plant growth, we were unable to demonstrate any effect of these compounds on potato plantlets grown in tissue culture.

The *budB* gene may contribute to *P. c.* subsp. *carotovorum* growth in plants directly by playing a role in nutrient and energy acquisition through fermentation. This fermentation pathway is not absolutely required for growth in potato, however, since the *budB* mutant still reached high cell densities in the area it was inoculated into potato. This pathway may contribute to virulence by alkalinizing the local environment, which favors pectate lyase activity. A related plant pathogen, *Serratia plymuthica*, grows well in carrot slices when the *budB* gene is deleted, thus, related pathogens also do not require this pathway for growth on plants [9]. Unlike *P. c.* subsp. *carotovorum*, *S. plymuthica* does not produce pectate lyases and it is a more aggressive plant pathogen when *budB* is deleted. Fungal pathogens, such as *Colletotrichum gloeosporioides*, also alkalinize their environment, which favors both expression and activity of fungal pectate lyases [64]. This convergence among bacterial and fungal pathogens that produce pectate lyases suggests that microbial ability to buffer the local environment is required for efficacy, and perhaps therefore selection and maintenance in the genome, of pectate lyases.

Some bacteria are able to utilize 3H2B and 23B as energy sources, but a search of the available *Pectobacterium* genomes in the ASAP database [65], [66] show that they lack the required genes, which were initially described in *Alcaligenes eutrophus* [67], as does the related soft rot pathogen *Dickeya* and the tree pathogen *Erwinia amylovora*. However, BLASTp searches show that these genes are widespread in other plant associated bacteria, including *Pseudomonas putida*, *Pseudomonas fluorescens*, *Erwinia tasmaniensis*, *Bacillus* species, and *Phytoplasma* species. It is also present in opportunistic human pathogens commonly found on plants, such as *Pseudomonas aeruginosa* and *Burkholderia cepacia*, although it is not in *Pseudomonas syringae*. It is possible that metabolism of 3H2B pathway products, which may buffer the environment for soft rot pathogens, would be counterproductive for these pathogens. There have been a few studies of co-colonization of soft rot bacterial pathogens with *S. enterica* or *E. coli* on plant leaves [68], [69], [70], which are bacteria that acidify their environment, and *Clostridium*, which can metabolize 3H2B are which are commonly found with *Pectobacterium* in decaying tubers [71]. The parameters that determine local pH in mixed infections of these types and whether it affects disease progress remain unknown.

The stability of a key *P. carotovorum* signal molecule, acyl-homoserine lactone (acyl-HSL), is also affected by pH and it is hydrolyzed into an inactive form above pH 6.8 [72]. As in many bacteria, acyl-HSL acts as a quorum sensing molecule in *Pectobacterium* and affects the regulation of approximately one quarter of *Pectobacterium* genes, including induction of both the *budAB* operon and plant cell wall degrading enzymes [60]. Since acyl-HSL hydrolyzes in alkaline pH, it should be less available once fermentation has started and the local pH has increased. Whether local concentrations of acyl-HSL are sufficient or other mechanisms, such as induction by plant cell wall fragments become more important in enzyme expression remains unknown.

The butanediol pathway has been examined in plant growth promoting bacteria (PGPB), where it is thought to be responsible for the growth promoting effects of some PGPB [22], [23]. A wide range of bacterial species commonly found in decaying plants, from *Pectobacterium* to *Bacillus subtilis* produce 3H2B and 23B through fermentation. If the growth promoting effects of 3H2B and 23B do occur, this suggests that plants may grow in response to this compound because it is a signal of bacterial decay of plants, and hence, a signal that a rich source of nutrients is available from the composting plants. To determine if 3H2B and 23B affect growth of potato, we added these compounds to tissue culture medium used to grow potato plantlets, but were unable to demonstrate that these compounds promote potato growth. Use of 3H2B and 23B as priming molecules for plant resistance and drought resistance has been suggested [23], [24], but this may be ineffective in practice since plants are likely already exposed to these molecules, which are produced by a wide range of soil microbes, on a daily basis.

The volatile products of the 3H2B pathway have long been known to attract a wide range of insects and it is common knowledge that a variety of insects are attracted to decaying plant material. For example, pest control recommendations for seed corn maggot (*Delia platura*) include avoiding plowing weeds, green manure or other cover crops in the spring since the adult female flies are attracted to decaying plants. A similar attraction to decaying onions has been seen with female, but not male, onion flies (*Delia antiqua*) [73]. Recently, Turner and Ray [19] found that 3H2B pathway volatiles suppress CO_2_ avoidance in *Drosophila*, which causes the flies to be attracted to ripening fruit. However, the connection that bacteria may attract insects specifically through the butanediol pathway has not yet been made. Insects are known vectors of soft rot bacteria [29], [30], [31], [32], [33], [34], [35], [36], [37], [38], [39], [41] and are also vectors of other closely related pathogens that also encode this pathway, such as *Erwinia amylovora* [74], providing support for a role of the 3H2B pathway in bacterial pathogen-insect symbiosis. Insects may benefit from this if the volatile compounds produced by the 3H2B pathway indicate a source of food (decaying plants) or a source of beneficial microbes that may aid them in invasion of plant hosts.

The Voges-Proskauer reaction is commonly used in introductory bacteriology laboratories to demonstrate biochemical methods used to identify bacteria, but then essentially ignored thereafter by many microbiologists. However, the butanediol pathway appears to play important roles in plant pathogenesis, insect behavior, and possible plant growth promotion. It may explain, in part, phenomena familiar to any gardener: insects are attracted to decaying plants and plants grow well in compost. It may also be an important step in evolution of soft rot pathogens, since acquisition of a pathway that alkalinizes the local environment may be required for maintenance of pectate lyases in pathogen genomes. Thus, this common diagnostic test is tied to important aspects of bacterial plant pathogenesis and evolution, and insect behavior.
